# 6-Hy­droxy-4-(pyridin-3-yl)-5-(2-thienyl­carbon­yl)-6-trifluoro­meth­yl-3,4,5,6-tetra­hydro­pyrimidin-2(1*H*)-one

**DOI:** 10.1107/S1600536810041085

**Published:** 2010-10-20

**Authors:** Gong-Chun Li, Zhi-Yu Ju, Hong-Sheng Wang, Yu-Jiao Niu, Feng-Ling Yang

**Affiliations:** a College of Chemistry and Chemical Engineering, Xuchang University, Xuchang, Henan Province, 461000, People’s Republic of China

## Abstract

In the title compound, C_15_H_12_F_3_N_3_O_3_S, the pyrimidine ring adopts a half-chair conformation with the mean plane formed by the ring atoms excluding the C atom bonded to thio­phene-2-carbonyl group lying nearly perpendicular to the pyridine and thio­phene rings, making dihedral angles of 84.91 (4) and 87.40 (5)°, respectively. The dihedral angle between the pyridine and thio­phene rings is 54.44 (5)°. The crystal structure is stabilized by inter­molecular O—H⋯O and N—H⋯N hydrogen bonds and weak C—H⋯O inter­actions further consolidate the structure.

## Related literature

For the bioactivity of dihydro­pyrimidines, see: Cochran *et al.* (2005 ▶); Moran *et al.* (2007 ▶); Zorkun *et al.* (2006 ▶). For the bioactivity of organofluorine compounds, see: Ulrich (2004 ▶). For a related stucture, see: Yang *et al.* (2009 ▶).
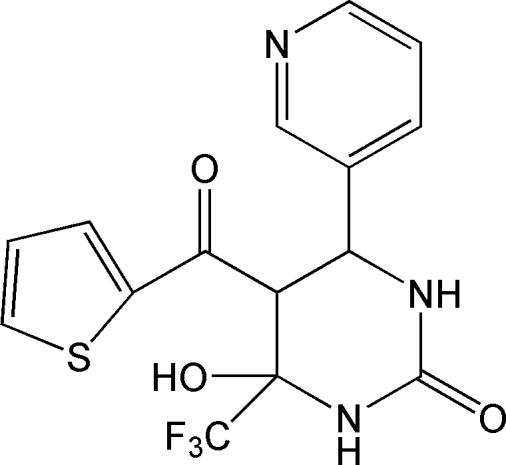


## Experimental

### 

#### Crystal data

C_15_H_12_F_3_N_3_O_3_S
*M* *_r_* = 371.34
Monoclinic, 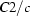

*a* = 29.694 (3) Å
*b* = 5.9710 (8) Å
*c* = 17.6910 (16) Å
β = 95.223 (8)°
*V* = 3123.6 (6) Å^3^
*Z* = 8
Mo *K*α radiation
μ = 0.26 mm^−1^
*T* = 113 K
0.26 × 0.22 × 0.20 mm

#### Data collection

Rigaku Saturn724 CCD diffractometer
Absorption correction: multi-scan (*CrystalClear-SM Expert*; Rigaku/MSC, 2009 ▶) *T*_min_ = 0.935, *T*_max_ = 0.950
15077 measured reflections
3707 independent reflections
2888 reflections with *I* > 2.0 σ(*I*)
*R*_int_ = 0.056

#### Refinement

*R*[*F* ^2^ > 2σ(*F* ^2^)] = 0.036
*wR*(*F* ^2^) = 0.098
*S* = 1.04
3707 reflections
238 parameters
H atoms treated by a mixture of independent and constrained refinement
Δρ_max_ = 0.36 e Å^−3^
Δρ_min_ = −0.23 e Å^−3^

### 

Data collection: *CrystalClear-SM Expert* (Rigaku/MSC, 2009 ▶); cell refinement: *CrystalClear-SM Expert*; data reduction: *CrystalClear-SM Expert*; program(s) used to solve structure: *SHELXS97* (Sheldrick, 2008 ▶); program(s) used to refine structure: *SHELXL97* (Sheldrick, 2008 ▶); molecular graphics: *CrystalStructure* (Rigaku/MSC, 2009 ▶); software used to prepare material for publication: *CrystalStructure*.

## Supplementary Material

Crystal structure: contains datablocks global, I. DOI: 10.1107/S1600536810041085/pv2334sup1.cif

Structure factors: contains datablocks I. DOI: 10.1107/S1600536810041085/pv2334Isup2.hkl

Additional supplementary materials:  crystallographic information; 3D view; checkCIF report

## Figures and Tables

**Table 1:** Hydrogen-bond geometry (Å, °)

*D*—H⋯*A*	*D*—H	H⋯*A*	*D*⋯*A*	*D*—H⋯*A*
O1—H5⋯O2^i^	0.81 (2)	1.94 (2)	2.7466 (14)	173 (2)
N2—H2⋯N3^ii^	0.85 (2)	2.21 (2)	3.0153 (15)	159 (2)
N1—H1⋯N3^iii^	0.87 (2)	2.19 (2)	3.0378 (16)	167 (2)
C3—H3⋯O2^i^	1.00	2.57	3.255 (2)	126
C7—H7⋯O3^iv^	0.95	2.49	3.108 (2)	123

Symmetry codes: (i) 
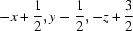
; (ii) 
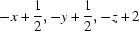
; (iii) 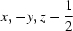; (iv) 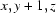.

**Fig. 1.**
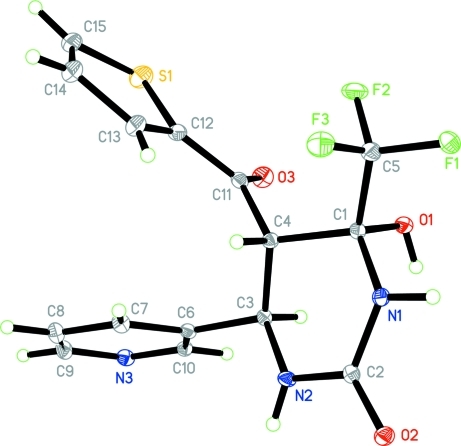
Molecular configuration and atom numbering scheme for (I), with displacement ellipsoids drawn at the 30% probability level.

**Fig. 2.**
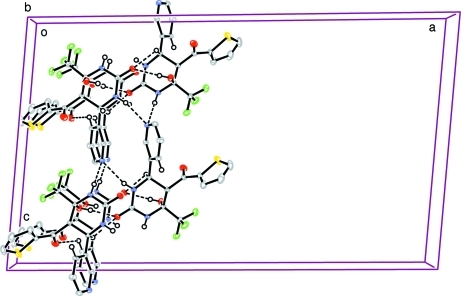
The packing diagram of the title compound.

## supplementary crystallographic information

### Comment

Dihydropyrimidine (DHPM) derivatives can be used as potential calcium channel blockers (Zorkun *et al.*, 2006), inhibitors of mitotic kinesin Eg5 (a member of the Kinesin-5 subclass of kinesins) for treating cancer (Cochran *et al.*, 2005) and as TRPA1 (Transient Receptor Potential cation channel, subfamily A, member 1) modulators for treating pain (Moran *et al.*, 2007). In addition, compounds that contain fluorine have special bioactivity, for example flumioxazin is a widely used herbicide (Ulrich, 2004). This led us to focus our attention on the synthesis and bioactivity of these important fused perfluoroalkylated heterocyclic compounds. During the synthesis of DHPM derivatives, the title compound, an intermediate (I) was isolated and its structure established by X-ray diffraction method, in order to elucidate the reaction mechanism.

In the structure of the title molecule, the dihydropyrimidine ring adopts a half-chair conformation; the atoms C1/C2/C3/N1/N2 are nearly coplanar and the plane is nearly perpendicular with pyridine and thiophene rings with dihedral angles 84.91 (4) and 87.40 (5)°, respectively. The dihedral angle between the pyridine ring and the thiophene ring is 54.44 (5)°. The crystal structure is stabilized by intermolecular hydrogen bonds of the types O—H···O and N—H···N; there are also weak hydrogen bonding interactions of the type C—H···O present in the structure (Table 1). For a crystal structure related to the title compound, see: Yang *et al.*, (2009).

### Experimental

The title compound was synthesized by refluxing for 3 h a stirred solution of 3-pyridinaldehyde (0.22 g, 2 mmol), 4,4,4-trifluoro-1-(thiophen-2-yl)butane- 1,3-dione (0.51 g, 2.3 mmol) and urea (0.18 g, 3 mmol) in 3 ml of anhydrous ethanol; the reaction was catalyzed by sulfamic acid (0.06 g). The solvent was evaporated *in vacuo* and the residue was washed with water. The title compound was recrystallized from 50% aqueous ethanol and single crystals of (I) were obtained by slow evaporation.

### Refinement

Hydrogen atoms involved in hydrogen-bonding inetractions were located from a difference Fourier map and their positional and isotropic displacement parameters were refined. The other H atoms were placed in calculated positions, with C—H(aromatic) = 0.95 Å and C—H(aliphatic) = 1.00 Å, and treated as riding, with *U*_iso_(H) = 1.2*U*_eq_(C).

### Crystal data

**Table:**

C_15_H_12_F_3_N_3_O_3_S	*F*(000) = 1520
*M**_r_* = 371.34	*D*_x_ = 1.579 Mg m^−^^3^
Monoclinic, *C*2/*c*	Mo *K*α radiation, λ = 0.71075 Å
Hall symbol: -C 2yc	Cell parameters from 5438 reflections
*a* = 29.694 (3) Å	θ = 1.7–28.0°
*b* = 5.9710 (8) Å	µ = 0.26 mm^−^^1^
*c* = 17.6910 (16) Å	*T* = 113 K
β = 95.223 (8)°	Prism, colorless
*V* = 3123.6 (6) Å^3^	0.26 × 0.22 × 0.20 mm
*Z* = 8	

### Data collection

**Table:**

Rigaku Saturn724 CCD diffractometer	3707 independent reflections
Radiation source: rotating anode	2888 reflections with *I* > 2.0 σ(*I*)
multilayer	*R*_int_ = 0.056
Detector resolution: 14.222 pixels mm^-1^	θ_max_ = 27.9°, θ_min_ = 2.3°
ω scans	*h* = −38→37
Absorption correction: multi-scan (*CrystalClear-SM Expert*; Rigaku/MSC, 2009)	*k* = −7→7
*T*_min_ = 0.935, *T*_max_ = 0.950	*l* = −23→23
15077 measured reflections	

### Refinement

**Table:**

Refinement on *F*^2^	Primary atom site location: structure-invariant direct methods
Least-squares matrix: full	Secondary atom site location: difference Fourier map
*R*[*F*^2^ > 2σ(*F*^2^)] = 0.036	Hydrogen site location: inferred from neighbouring sites
*wR*(*F*^2^) = 0.098	H atoms treated by a mixture of independent and constrained refinement
*S* = 1.04	*w* = 1/[σ^2^(*F*_o_^2^) + (0.0496*P*)^2^] where *P* = (*F*_o_^2^ + 2*F*_c_^2^)/3
3707 reflections	(Δ/σ)_max_ = 0.001
238 parameters	Δρ_max_ = 0.36 e Å^−^^3^
0 restraints	Δρ_min_ = −0.23 e Å^−^^3^

### Special details

**Table:**

Geometry. All e.s.d.'s (except the e.s.d. in the dihedral angle between two l.s. planes) are estimated using the full covariance matrix. The cell e.s.d.'s are taken into account individually in the estimation of e.s.d.'s in distances, angles and torsion angles; correlations between e.s.d.'s in cell parameters are only used when they are defined by crystal symmetry. An approximate (isotropic) treatment of cell e.s.d.'s is used for estimating e.s.d.'s involving l.s. planes.
Refinement. Refinement of *F*^2^ against ALL reflections. The weighted *R*-factor *wR* and goodness of fit *S* are based on *F*^2^, conventional *R*-factors *R* are based on *F*, with *F* set to zero for negative *F*^2^. The threshold expression of *F*^2^ > σ(*F*^2^) is used only for calculating *R*-factors(gt) *etc*. and is not relevant to the choice of reflections for refinement. *R*-factors based on *F*^2^ are statistically about twice as large as those based on *F*, and *R*- factors based on ALL data will be even larger.

### Fractional atomic coordinates and isotropic or equivalent isotropic displacement parameters (Å^2^)

**Table:**

	*x*	*y*	*z*	*U*_iso_*/*U*_eq_	
S1	0.026595 (14)	0.12707 (8)	0.92814 (2)	0.03528 (14)	
F1	0.10963 (3)	−0.11784 (17)	0.62064 (4)	0.0313 (2)	
F2	0.06951 (3)	−0.11496 (17)	0.71617 (5)	0.0298 (2)	
F3	0.09202 (3)	0.19531 (16)	0.67005 (5)	0.0302 (2)	
O1	0.15452 (3)	−0.25213 (17)	0.75794 (5)	0.0181 (2)	
O2	0.25389 (3)	0.21588 (17)	0.70458 (5)	0.0176 (2)	
O3	0.10702 (3)	−0.12933 (18)	0.89515 (5)	0.0238 (2)	
N1	0.18320 (4)	0.0743 (2)	0.70261 (6)	0.0155 (3)	
N2	0.21958 (4)	0.2166 (2)	0.81451 (6)	0.0153 (3)	
N3	0.19968 (4)	0.2310 (2)	1.07057 (6)	0.0179 (3)	
C1	0.14728 (4)	−0.0243 (2)	0.74123 (7)	0.0146 (3)	
C2	0.22112 (4)	0.1687 (2)	0.74020 (7)	0.0143 (3)	
C3	0.18652 (4)	0.1181 (2)	0.86052 (7)	0.0136 (3)	
H3	0.1952	−0.0410	0.8719	0.016*	
C4	0.14098 (4)	0.1215 (2)	0.81197 (7)	0.0141 (3)	
H4	0.1345	0.2788	0.7949	0.017*	
C5	0.10424 (5)	−0.0160 (3)	0.68637 (7)	0.0211 (3)	
C6	0.18514 (4)	0.2436 (2)	0.93467 (7)	0.0144 (3)	
C7	0.16718 (4)	0.4565 (3)	0.93907 (7)	0.0178 (3)	
H7	0.1565	0.5349	0.8943	0.021*	
C8	0.16500 (5)	0.5540 (3)	1.01013 (7)	0.0208 (3)	
H8	0.1524	0.6990	1.0148	0.025*	
C9	0.18153 (5)	0.4355 (3)	1.07377 (7)	0.0201 (3)	
H9	0.1799	0.5025	1.1222	0.024*	
C10	0.20114 (4)	0.1383 (2)	1.00190 (7)	0.0153 (3)	
H10	0.2138	−0.0072	0.9990	0.018*	
C11	0.10371 (4)	0.0461 (3)	0.85989 (7)	0.0168 (3)	
C12	0.06578 (5)	0.1986 (3)	0.86639 (7)	0.0206 (3)	
C13	0.05608 (5)	0.4038 (3)	0.83352 (9)	0.0255 (3)	
H13	0.0738	0.4723	0.7978	0.031*	
C14	0.01670 (5)	0.5012 (3)	0.85897 (10)	0.0372 (4)	
H14	0.0049	0.6425	0.8423	0.045*	
C15	−0.00227 (5)	0.3696 (3)	0.90984 (10)	0.0404 (5)	
H15	−0.0289	0.4088	0.9328	0.048*	
H1	0.1888 (5)	0.007 (3)	0.6611 (9)	0.024 (4)*	
H2	0.2439 (5)	0.260 (3)	0.8390 (9)	0.020 (4)*	
H5	0.1814 (6)	−0.273 (3)	0.7674 (10)	0.031 (5)*	

### Atomic displacement parameters (Å^2^)

**Table:**

	*U*^11^	*U*^22^	*U*^33^	*U*^12^	*U*^13^	*U*^23^
S1	0.0254 (2)	0.0467 (3)	0.0363 (2)	−0.00599 (19)	0.01643 (16)	−0.00842 (19)
F1	0.0228 (4)	0.0510 (7)	0.0189 (4)	0.0021 (4)	−0.0042 (3)	−0.0141 (4)
F2	0.0159 (4)	0.0449 (7)	0.0283 (5)	−0.0080 (4)	0.0001 (3)	−0.0047 (4)
F3	0.0265 (5)	0.0355 (6)	0.0266 (4)	0.0091 (4)	−0.0088 (3)	0.0028 (4)
O1	0.0159 (5)	0.0168 (6)	0.0216 (5)	−0.0013 (4)	0.0015 (4)	−0.0016 (4)
O2	0.0170 (5)	0.0202 (6)	0.0161 (4)	−0.0023 (4)	0.0039 (3)	0.0002 (4)
O3	0.0260 (5)	0.0226 (6)	0.0233 (5)	−0.0020 (4)	0.0059 (4)	0.0052 (4)
N1	0.0154 (5)	0.0206 (7)	0.0107 (5)	−0.0008 (5)	0.0019 (4)	−0.0017 (4)
N2	0.0140 (6)	0.0198 (7)	0.0121 (5)	−0.0037 (5)	0.0005 (4)	−0.0012 (4)
N3	0.0187 (6)	0.0215 (7)	0.0133 (5)	−0.0030 (5)	0.0008 (4)	0.0005 (5)
C1	0.0137 (6)	0.0152 (8)	0.0147 (6)	0.0003 (5)	0.0002 (4)	−0.0008 (5)
C2	0.0163 (6)	0.0119 (7)	0.0146 (6)	0.0006 (5)	0.0005 (5)	0.0010 (5)
C3	0.0150 (6)	0.0140 (7)	0.0119 (6)	−0.0006 (5)	0.0013 (4)	0.0006 (5)
C4	0.0135 (6)	0.0150 (8)	0.0137 (6)	0.0001 (5)	0.0011 (5)	0.0003 (5)
C5	0.0180 (7)	0.0274 (9)	0.0176 (6)	0.0000 (6)	−0.0003 (5)	−0.0035 (6)
C6	0.0143 (6)	0.0151 (8)	0.0137 (6)	−0.0026 (6)	0.0016 (4)	−0.0005 (5)
C7	0.0209 (7)	0.0178 (8)	0.0146 (6)	−0.0008 (6)	0.0011 (5)	0.0015 (5)
C8	0.0257 (7)	0.0166 (8)	0.0201 (6)	−0.0006 (6)	0.0031 (5)	−0.0027 (5)
C9	0.0237 (7)	0.0222 (9)	0.0146 (6)	−0.0025 (6)	0.0029 (5)	−0.0033 (5)
C10	0.0144 (6)	0.0162 (8)	0.0155 (6)	−0.0013 (5)	0.0024 (5)	0.0008 (5)
C11	0.0165 (6)	0.0191 (8)	0.0147 (6)	−0.0028 (6)	0.0009 (5)	−0.0023 (5)
C12	0.0152 (7)	0.0270 (9)	0.0197 (6)	−0.0026 (6)	0.0024 (5)	−0.0057 (6)
C13	0.0197 (7)	0.0257 (9)	0.0307 (8)	0.0054 (7)	0.0006 (6)	−0.0044 (6)
C14	0.0235 (8)	0.0365 (12)	0.0499 (10)	0.0109 (8)	−0.0060 (7)	−0.0172 (8)
C15	0.0178 (8)	0.0559 (14)	0.0483 (10)	−0.0007 (8)	0.0075 (7)	−0.0310 (9)

### Geometric parameters (Å, °)

**Table:**

S1—C15	1.699 (2)	C3—C4	1.5345 (17)
S1—C12	1.7208 (15)	C3—H3	1.0000
F1—C5	1.3347 (15)	C4—C11	1.5222 (17)
F2—C5	1.3379 (16)	C4—H4	1.0000
F3—C5	1.3370 (18)	C6—C7	1.383 (2)
O1—C1	1.4047 (17)	C6—C10	1.3902 (17)
O1—H5	0.81 (2)	C7—C8	1.3922 (18)
O2—C2	1.2390 (16)	C7—H7	0.9500
O3—C11	1.2187 (17)	C8—C9	1.3813 (19)
N1—C2	1.3753 (17)	C8—H8	0.9500
N1—C1	1.4435 (17)	C9—H9	0.9500
N1—H1	0.87 (2)	C10—H10	0.9500
N2—C2	1.3501 (16)	C11—C12	1.461 (2)
N2—C3	1.4553 (16)	C12—C13	1.375 (2)
N2—H2	0.85 (2)	C13—C14	1.416 (2)
N3—C9	1.3379 (19)	C13—H13	0.9500
N3—C10	1.3393 (16)	C14—C15	1.355 (3)
C1—C5	1.5337 (18)	C14—H14	0.9500
C1—C4	1.5495 (17)	C15—H15	0.9500
C3—C6	1.5145 (17)		
			
C15—S1—C12	91.49 (9)	F1—C5—C1	112.19 (11)
C1—O1—H5	108.7 (13)	F3—C5—C1	111.18 (12)
C2—N1—C1	123.09 (10)	F2—C5—C1	111.36 (11)
C2—N1—H1	112.9 (10)	C7—C6—C10	118.08 (12)
C1—N1—H1	114.8 (11)	C7—C6—C3	122.98 (11)
C2—N2—C3	122.91 (11)	C10—C6—C3	118.90 (12)
C2—N2—H2	117.3 (11)	C6—C7—C8	119.02 (12)
C3—N2—H2	115.1 (11)	C6—C7—H7	120.5
C9—N3—C10	117.51 (11)	C8—C7—H7	120.5
O1—C1—N1	112.90 (10)	C9—C8—C7	118.65 (14)
O1—C1—C5	105.49 (11)	C9—C8—H8	120.7
N1—C1—C5	107.24 (10)	C7—C8—H8	120.7
O1—C1—C4	113.67 (10)	N3—C9—C8	123.19 (12)
N1—C1—C4	107.58 (11)	N3—C9—H9	118.4
C5—C1—C4	109.76 (11)	C8—C9—H9	118.4
O2—C2—N2	123.02 (12)	N3—C10—C6	123.54 (13)
O2—C2—N1	119.57 (11)	N3—C10—H10	118.2
N2—C2—N1	117.31 (12)	C6—C10—H10	118.2
N2—C3—C6	110.93 (11)	O3—C11—C12	121.48 (13)
N2—C3—C4	106.64 (10)	O3—C11—C4	120.69 (12)
C6—C3—C4	112.73 (11)	C12—C11—C4	117.68 (13)
N2—C3—H3	108.8	C13—C12—C11	130.93 (13)
C6—C3—H3	108.8	C13—C12—S1	111.21 (11)
C4—C3—H3	108.8	C11—C12—S1	117.77 (11)
C11—C4—C3	109.42 (10)	C12—C13—C14	112.26 (16)
C11—C4—C1	115.54 (11)	C12—C13—H13	123.9
C3—C4—C1	106.24 (10)	C14—C13—H13	123.9
C11—C4—H4	108.5	C15—C14—C13	112.22 (17)
C3—C4—H4	108.5	C15—C14—H14	123.9
C1—C4—H4	108.5	C13—C14—H14	123.9
F1—C5—F3	107.07 (11)	C14—C15—S1	112.82 (13)
F1—C5—F2	107.45 (12)	C14—C15—H15	123.6
F3—C5—F2	107.35 (11)	S1—C15—H15	123.6
			
C2—N1—C1—O1	−88.51 (15)	N2—C3—C6—C7	71.30 (16)
C2—N1—C1—C5	155.73 (13)	C4—C3—C6—C7	−48.22 (17)
C2—N1—C1—C4	37.72 (17)	N2—C3—C6—C10	−111.02 (13)
C3—N2—C2—O2	−165.46 (13)	C4—C3—C6—C10	129.46 (13)
C3—N2—C2—N1	18.18 (19)	C10—C6—C7—C8	−1.32 (19)
C1—N1—C2—O2	167.89 (12)	C3—C6—C7—C8	176.37 (12)
C1—N1—C2—N2	−15.6 (2)	C6—C7—C8—C9	1.0 (2)
C2—N2—C3—C6	−165.76 (12)	C10—N3—C9—C8	−0.8 (2)
C2—N2—C3—C4	−42.66 (17)	C7—C8—C9—N3	0.1 (2)
N2—C3—C4—C11	−173.34 (11)	C9—N3—C10—C6	0.38 (19)
C6—C3—C4—C11	−51.37 (15)	C7—C6—C10—N3	0.7 (2)
N2—C3—C4—C1	61.28 (13)	C3—C6—C10—N3	−177.13 (12)
C6—C3—C4—C1	−176.75 (11)	C3—C4—C11—O3	−52.53 (17)
O1—C1—C4—C11	−55.20 (14)	C1—C4—C11—O3	67.28 (16)
N1—C1—C4—C11	179.03 (10)	C3—C4—C11—C12	123.10 (12)
C5—C1—C4—C11	62.66 (15)	C1—C4—C11—C12	−117.09 (13)
O1—C1—C4—C3	66.33 (13)	O3—C11—C12—C13	178.48 (14)
N1—C1—C4—C3	−59.43 (13)	C4—C11—C12—C13	2.9 (2)
C5—C1—C4—C3	−175.80 (11)	O3—C11—C12—S1	2.12 (18)
O1—C1—C5—F1	−64.29 (14)	C4—C11—C12—S1	−173.47 (9)
N1—C1—C5—F1	56.29 (16)	C15—S1—C12—C13	0.00 (12)
C4—C1—C5—F1	172.87 (12)	C15—S1—C12—C11	177.05 (11)
O1—C1—C5—F3	175.84 (10)	C11—C12—C13—C14	−176.56 (14)
N1—C1—C5—F3	−63.58 (14)	S1—C12—C13—C14	−0.02 (16)
C4—C1—C5—F3	53.01 (15)	C12—C13—C14—C15	0.03 (19)
O1—C1—C5—F2	56.18 (14)	C13—C14—C15—S1	−0.04 (18)
N1—C1—C5—F2	176.76 (11)	C12—S1—C15—C14	0.02 (13)
C4—C1—C5—F2	−66.65 (15)		

### Hydrogen-bond geometry (Å, °)

**Table:**

*D*—H···*A*	*D*—H	H···*A*	*D*···*A*	*D*—H···*A*
O1—H5···O2^i^	0.81 (2)	1.94 (2)	2.7466 (14)	173 (2)
N2—H2···N3^ii^	0.85 (2)	2.21 (2)	3.0153 (15)	159 (2)
N1—H1···N3^iii^	0.87 (2)	2.19 (2)	3.0378 (16)	167 (2)
C3—H3···O1	1.00	2.58	2.961 (2)	102
C3—H3···O2^i^	1.00	2.57	3.255 (2)	126
C7—H7···O3^iv^	0.95	2.49	3.108 (2)	123

Symmetry codes: (i) −*x*+1/2, *y*−1/2, −*z*+3/2; (ii) −*x*+1/2, −*y*+1/2, −*z*+2; (iii) *x*, −*y*, *z*−1/2; (iv) *x*, *y*+1, *z*.
